# Reforming the culture of healthcare: the case for intelligent kindness

**DOI:** 10.1192/pb.bp.114.047449

**Published:** 2015-02

**Authors:** Penelope Campling

**Affiliations:** 1 Retired, formerly a clinical director at the Leicestershire Partnership Trust

## Abstract

There has been increasing interest in the culture of healthcare in the light of the two reports by Robert Francis into the care at Mid Staffordshire. This editorial encourages a comprehensive exploration of the conditions that promote a benign caring culture and make outbreaks of cruel neglect and abuse of patients less likely. Creating and sustaining such a culture is dependent on being honest and realistic about the forces that threaten to undermine it. The editorial argues that being able to confidently articulate the positive values that should define healthcare culture is particularly important at this time. The case is made for a conscious focus on the concept of intelligent kindness.

Robert Francis’ two reports into the Mid Staffordshire scandal,^1,2^ other inquiry reports such as Winterbourne View^3^ and, most recently, Julia Neuberger’s inquiry into the Liverpool Care Pathway^4^ face us with the reality that healthcare organisations and the healthcare workers within them are capable of neglectful and abusive behaviour that can justifiably be described as cruel. The outrage presses towards sackings and resignations, to a ‘never again’ attitude, or, in various quarters, towards further denigration of the National Health Service (NHS) as a public enterprise. The determination to do something directs us towards yet more regulation and hundreds of other initiatives. Many of these actions may make a difference, but there is a risk that they will become yet another ‘programme’ of activity, on top of an already toppling tower of initiatives, and vulnerable to becoming a mechanical activity rather than keeping their purpose (securing kindness and compassion) in mind.

There has been a general call to address the culture of the NHS. Again, there are signs that this will become another programme – split off from or, at best, sitting alongside, others like the drive for efficiency, promoting choice, etc., rather than a comprehensive consideration of the totality of the conditions in which the NHS works, a genuine exploration of how the NHS culture promotes kindness, or cruelty.

As part of this enterprise, this editorial makes the case for a more conscious and active focus on the concept of intelligent kindness in all parts of the healthcare system. It starts, however, by exploring the forces that create perverse dynamics that can pull in the opposite direction.

## The emotional task

Why do seemingly caring staff behave unkindly? This question can be looked at from many perspectives, starting at the level of the individual.^5^ While it is clearly important to think carefully about recruitment criteria and to encourage people to think clearly about their motivation to work in healthcare, studies suggest that the majority of healthcare students are motivated by the wish to make things better, but during their training become more distanced from patients and less empathic.^6,7^

Raymond Tallis, British philosopher and retired professor of geriatric medicine, comments on the enormity in the history of civilisation of the imaginative and moral step involved in engaging with the realities of illness.^8^ He describes a challenging process of cognitive self-overcoming on the part of humanity and reminds us that humans found it easier to assume an objective attitude towards the stars than towards their own inner organs. This self-overcoming – surely one of humanity’s greatest achievements – has to be done on an individual level by thousands of NHS staff every day as they muster the will, the necessary balance of kindness and professional detachment, to perform the most intimate tasks imaginable. It is easy to forget the appalling nature of some of the jobs carried out by healthcare staff day in, day out – the damage, the pain, the mess they encounter, the sheer stench of diseased human flesh and its waste products.

Contact with emotional distress and disturbance can be equally, if not more, harrowing. Existential questions about identity, suffering, madness and death are raised and may put people in touch with extreme feelings of confusion, pain and loss.^9^ The struggle with feelings of helplessness and hopelessness in the face of suffering cannot be avoided, and individuals, depending on their personality and past experience, protect themselves in different ways from the emotionally traumatic environment.^10^ Psychological defence mechanisms are evoked frequently. Problems can arise if staff are exposed to frequent emotional trauma, without space to process their feelings. Defensive styles of coping then become entrenched.^11^ As defensive walls build up, feelings of vulnerability and sadness become more deeply buried and the capacity for empathy recedes.

## Problematic team-working

Attachment to a well-functioning group can help to contain disturbed feelings and facilitate a healthy focus on the emotional task, but groups can also be the scene of disturbed – often unconscious – dynamics.^12^ There is evidence that the quality of the team one is working within makes a lot of difference to staff experience, can buffer the effects of a wider dysfunctional organisation^1^ and enhances functioning generally.^13^ Unfortunately, many hospital teams are not highly evolved in their functioning as teams. They tend to have unclear boundaries and conflicting objectives, with different professions approaching the task from different perspectives and tensions sometimes arising between professional and organisational hierarchies. In addition, many staff are on rapid training rotations or can be moved without consultation to cover shortages in other teams. The breakdown of close-knit medical ‘firms’ means patients often complain that they see a series of junior doctors and do not know the name of their consultant. Many staff, particularly senior staff, have a peripatetic role and belong in many different ‘teams’. Although most hospital staff say they belong to a team, Borrill *et al*^14^ show that more than 60% are in what they call pseudo-teams, with no obvious cohesion or boundary. Responsibility-shifting, driven by fending off anxiety between team members or between teams in wider system, is a particularly prevalent dynamic in health and social care.^15,16^

## Problematic organisations

Menzies Lyth’s famous study of nurses in the 1950s sought to understand why nurses resigned from their profession in such high numbers.^17^ It showed that the stresses of nursing, and the intimate relationship it demanded with patients, made an impact on the organisation of care, leaving those closest to patients exposed to emotional pressures that most senior staff and managers were defended against. Menzies Lyth felt that the work of nursing – what she called the objective situation – because it involves physical and emotional contact with illness, pain, suffering and death, arouses feelings and associated thoughts linked to the deepest and most primitive levels of the mind. She proceeded to show how the organisation of the hospital can be seen as consciously and unconsciously structured around the evasion of this anxiety.

Menzies Lyth proposed that the success and viability of a social institution are intimately connected with the techniques it uses to contain anxiety. In the intervening years, these ideas have been developed, looking at the goodness of fit between organisation structures on the one hand, and the emotional demands of healthcare work on the other. There is little sign that the system as a whole has developed effective structures to support frontline staff process the emotional disturbance inherent in their interactions with sick patients; in fact, evidence from the annual staff surveys suggests the opposite (www.nhsstaffsurveys.com). Moreover, there is little understanding or attempt to contain the primitive anxieties that pervade the system and affect all involved, including decision makers at government level. If anything, there is more disconnection between the policy level of the organisation and the emotional reality of clinical encounters.^18^

Whereas much of Menzies Lyth’s 1959 study could be describing the health service of today, there is one important difference. Menzies Lyth noted the resistance to change in the NHS of the 1950s and saw it as a significant part of the social defence system. I suggest here that it is the uncritical promotion of constant change and imposition of new ideologies that is the main social defence system in the modern health service,^19^ overloading and fragmenting the system and distracting from the task of caring for the sick and dying.^20–23^

## Perverse dynamics

The health service sits within a broader society that shapes its rules, agreements and unconscious social pacts. The spirit of cooperation that was around in the immediate aftermath of the Second World War provided a fertile value base for implementing the NHS, but has been steadily encroached upon by individualism, consumerism and the hegemony of market forces. Susan Long describes and gives evidence for this in her book *The Perverse Organisation and Its Deadly Sins*.^24^ A basic premise of her book is that there has been a move in society generally from a culture of narcissism to elements of a culture of perversion. Perversion flourishes where instrumental relations have dominance – in other words, where people are used as a means to an end, as tools and commodities rather than respected citizens. It is these relations that Long sees predominating increasingly. Her book considers large private corporations rather than the public sector. However, the fashion to idealise large private sector corporations and the subsequent corporatisation of the public sector means much of the thinking in her book is relevant to the modern health service.

It is important to realise that Long’s emphasis is on perversity displayed by institutions rather than by their leaders or members. There is no suggestion that individual NHS workers, as people, are any more perverse than workers in any other organisation. Nevertheless, in reality, an organisation and its members are entwined: the decisions and actions of individuals are influenced by organisational culture and, in turn, reinforce it, for good or ill. The concept of perversion sheds light on frankly exploitative behaviour, helps explain how many people in positions of trust end up abusing those positions and how people may be collectively perverse despite individual attempts to be otherwise.

## Corrupting forces?

There appear to be four closely intertwined processes at work. None of them is perverse in itself, but separately and together they can create perverse dynamics in the context of healthcare. The first is the active promotion of a competitive market economy, on the basis of a commodified view of need, skills and service. Such an economy works against the idea of an integrated service that prioritises the needs of vulnerable patients, and can insidiously affect the attitudes, feelings and relationships of staff.^25,26^ The second is the process of industrialising healthcare.^27,28^ This enterprise has the potential to undermine healthcare as work undertaken by skilled individuals in relationships with patients and to turn it into the mechanical delivery of processes and systems. The third is the framework and currency of specification, regulation and performance management. How services are specified, monitored and evaluated – and funded – has a profound effect on the day-to-day clinical work.^29–31^ The fourth is the inexorable rise of consumerism and the promotion of patient ‘choice’. These four elements are of course interrelated and, some would say, reflect inevitable trends in society at large. But of particular concern is the way these processes have taken hold without proper debate and understanding of the unintended consequences for the system as a whole.

## Focusing on compassion and kindness

In the light of the present crisis in the culture of our healthcare system, it is particularly important to be able to talk in terms of positive values, to have a clear vision of how we would like to see our organisations function, how we wish to encourage society – and the organisations that serve society – to relate to the sick and vulnerable. The NHS was founded at a particular point in history when there was a strong motivation to create a better future based on the idea of the common good – a concept that may be out of fashion but is still enshrined in the NHS constitution.^32^

If our public organisations are to flourish, we need to be able to articulate our aspirations in ways that resonate with today’s citizens. A number of writers and philosophers have attempted to address the worrying narrowing of the moral universe in organisational life: Paul Ricoeur refers to the loss of ethical intention in public life;^33^ Onora O’Neil talks about the growing culture of suspicion linked to increasingly excessive accountability regimes and urges us to free professionals and their public services to serve the public;^34^ Michael Sandel talks about the squeezing out of altruism and argues that we put limits on the current encroachment of market thinking into every sphere of life;^35^ and Tony Judt made an appeal before he died that we rediscover a language around which we can be motivated collectively, whether on the issue of justice, inequality, cruelty or unethical behaviour – a language that will bind us together.^36,37^

There has been a focus recently on compassion in healthcare.^38,39^ Although the popular press tends to see this as a nursing issue, there is wider acknowledgement that creating a more compassionate culture will need a systemic approach. There has been a growing interest more generally in compassionate leadership and the ‘compassionate organisation’ (www.compassionateleadership.com; http://instituteforcompassionateleadership.org).^40^

It is clear – and understandable from an evolutionary perspective – that if a person is feeling under threat, it is likely that the compassionate components of the mind are turned off and instead the mind has a pattern of motivation and ways of feeling that are about protecting oneself from danger. This is of obvious relevance to the NHS workforce and points to the creation of a culture that feels safe and affirming rather than unsettled and threatening.^41^

Clearly, there is a large overlap between the concept of compassion and the concept of kindness. Both words are defined in relation to other people: compassion literally meaning ‘suffering with’ whereas kindness is linked to the concept of kin and kinship. Kindness is a word very commonly used by patients. Many people’s stories about their experience of healthcare centre around the degree and quality of kindness they have (or have not) experienced. Often these accounts are complaints about the absence of kindness, the thoughtlessness, the lack of humane care. Sometimes they describe the power of small, but highly relevant, acts of kindness to transform an otherwise miserable experience of suffering (www.patientopinion.org.uk).^42,43^

Kindness is a word with an interesting history. It is also a word that needs rescuing for it can evoke mixed feelings in the modern world and easily become a mere synonym for individual acts of generosity, sentiment and affection, for a general fuzzy ‘kindliness’. The warping and obscuring of what kindness is about have been extensively discussed by psychoanalyst Adam Philips and historian Barbara Taylor in their recent book, *On Kindness*.^44^ They explore the way in which a philosophy and culture of competitive individualism and the pursuance of self-interest has challenged the value, and negatively influenced the meaning, of kindness. Kindness, they say, is not a temptation to sacrifice ourselves, but to include ourselves with others – kindness is being in solidarity with human need. They describe a process in which what had been a core moral value, with a subversive edge, at centre stage in the political battles of the Enlightenment, became something sentimentalised, marginalised and denigrated through the 19th and into the early 20th century.

## ‘Intelligent kindness’

Kindness has its roots in the Old English word *cynd* – meaning nature, family, lineage – kin. Kindness implies the recognition of being of the same nature as others, being of a kind, in kinship. It implies that people are motivated by that recognition to cooperate, to treat others as members of the family, to be generous and thoughtful. The word can be understood at an individual and at a collective level, and from an emotional, cognitive, even political point of view. Adding the adjective ‘intelligent’ signals, first that it is possible to think in a sophisticated way about the conditions for kindness, and second that clinical, managerial, leadership and organisational skills and systems can be brought to bear purposively to promote compassionate care. Intelligent kindness, then, is not a soft, sentimental feeling or action that is beside the point in the challenging, clever, technical business of managing and delivering healthcare. It is a binding, creative and problem-solving force that inspires and focuses the imagination and goodwill. It inspires and directs the attention and efforts of people and organisations towards building relationships with patients, recognising their needs and treating them well. Kindness is not a ‘nice’ side issue in the project of competitive progress. It is the ‘glue’ of cooperation required for such progress to be of most benefit to most people.

**Fig. 1:**
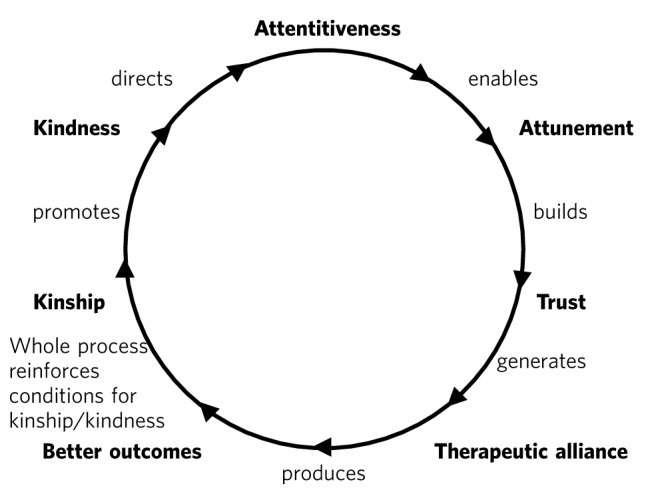
Intelligent kindness: a virtuous circle.

To illustrate how such behaviour is nurtured in the wider system a virtuous circle is envisaged, where there is not only a compassionate connection between the clinician and the patient, but the potential for something to happen in the wider system (Fig. 1).

There is a body of evidence that supports this virtuous circle, cited elsewhere.^45^ Simply put, the more attentively kind staff are, the more their attunement to the patient increases; the more that increases, the more trust is generated; the more trust, the better the therapeutic alliance; the better the alliance, the better the outcomes. The result of all this is a reduction in anxiety, improved satisfaction (for staff and patient), less defensiveness and improved conditions for kindness. This system will flourish if individuals and the system as a whole are driven by a sense of kinship. This can be expressed as simply as seeing oneself in the patient – or as the King’s Fund put it, seeing the person in the patient and delivering the sort of care you would like for your family and friends.^46^ This sense of kinship will promote the feeling and expression of kindness which then directs attention, and so on.

These dynamic processes can also contribute to productivity, a key challenge for all health services. A useful concept in the industrial model is that of ‘getting it right first time’ as a key driver for eliminating waste – of

time and resources. All stages and the combined effect of this cycle contribute to such effective activity. The more work is founded on kinship, motivated by kindness and expressed through attentiveness and attunement to the patient’s needs, the more it is likely to be timely and ‘right first time’.

## Conclusion

Kindness rooted in kinship is a powerful concept – ethically, politically, socially and clinically – in the project of improving healthcare. It increases patient satisfaction, staff morale, clinical effectiveness and efficiency. But virtuous circles are vulnerable and we know from history how quickly a benign culture can become malignant. The first part of this editorial described some of the difficulties inherent in the healthcare task that make a benign culture difficult to sustain if they are not properly understood and managed.

Menzies Lyth’s work on social defence systems in healthcare was published over 50 years ago. In general, though, there has been a failure to create organisations that are fit for purpose and able to facilitate the emotional work that is such an important component of the healthcare task. There has been a failure to acknowledge and get to grips with the way overwhelming anxiety – largely unconscious – can unhelpfully drive and undermine the system. Moreover, it is suggested that some of the changes in society over this time period have had an impact on the health service in a way that has amplified the amount of anxiety in the system, pulling the culture in a direction where perverse behaviours become more likely. Many would say the system has already become a vicious circle where so-called ‘solutions’ involve overloading the system and creating ever more dangerous levels of anxiety. Virtuous circles unravel so easily; vicious circles, on the other hand, are extremely difficult to break.

It is more important than ever to have an explicit value base underpinning the work of both individual staff members and healthcare organisations, and to understand what that value base looks like ‘in action’. The virtuous circle described here earlier could provide a basis for thinking about this, strengthening relationships between colleagues and with patients, and counteracting the pressures to adopt instrumental attitudes to the work that are all too prevalent at the present time. The possibility emerges of a kinder culture developing as all aspects of the NHS – evidence, skill, new technologies, where money is spent, how people are managed – are scrutinised in terms of how they support this virtuous circle.

At an anecdotal level, individuals report that the concept of intelligent kindness properly embedded in reflective practice has ‘reconnected them to their altruism’; and teams from ward to board level have found the virtuous circle a helpful focus when thinking about culture change. There is scope for adapting the model for research and audit purposes, building on the evidence base for relational science to influence the organisation of healthcare delivery and outcome.
